# Antibiotic resistance: What is so special about multidrug-resistant Gram-negative bacteria?

**DOI:** 10.3205/dgkh000290

**Published:** 2017-04-10

**Authors:** Martin Exner, Sanjay Bhattacharya, Bärbel Christiansen, Jürgen Gebel, Peter Goroncy-Bermes, Philippe Hartemann, Peter Heeg, Carola Ilschner, Axel Kramer, Elaine Larson, Wolfgang Merkens, Martin Mielke, Peter Oltmanns, Birgit Ross, Manfred Rotter, Ricarda Maria Schmithausen, Hans-Günther Sonntag, Matthias Trautmann

**Affiliations:** 1 Institute of Hygiene and Public Health, Bonn University, Bonn, Germany; 2 Tata Medical Center, Kolkata, India; 3 Department of Internal Hygiene, Schleswig-Holstein University Hospital, Kiel, Germany; 4 Schülke & Mayr GmbH, Norderstedt, Germany; 5 Departement Environnement et Santé Publique S.E.R.E.S., Faculté de Médecine, Nancy, France; 6 Institute of Medical Microbiology and Hygiene, University of Tübingen, Germany; 7 Institute of Hygiene and Environmental Medicine, University Medicine Greifswald, Germany; 8 School of Nursing, Columbia University, New York, USA; 9 Mailman School of Public Health, Columbia University, New York, USA; 10 Robert Koch Institute (RKI), Berlin, Germany; 11 Hospital Hygiene, Essen University Hospital, Essen, Germany; 12 Hygiene Institute, Medical University Vienna, Austria; 13 Institute of Hygiene and Medical Microbiology, University of Heidelberg, Germany; 14 Department of Hospital Hygiene, Stuttgart Hospital, Stuttgart, Germany

**Keywords:** multidrug-resistant Gram-negative bacteria, epidemiology, surveillance, reservoirs, resistance patterns, therapy, infection control measures, biocides, disinfection, agriculture

## Abstract

In the past years infections caused by multidrug-resistant Gram-negative bacteria have dramatically increased in all parts of the world. This consensus paper is based on presentations, subsequent discussions and an appraisal of current literature by a panel of international experts invited by the Rudolf Schülke Stiftung, Hamburg. It deals with the epidemiology and the inherent properties of Gram-negative bacteria, elucidating the patterns of the spread of antibiotic resistance, highlighting reservoirs as well as transmission pathways and risk factors for infection, mortality, treatment and prevention options as well as the consequences of their prevalence in livestock. Following a global, One Health approach and based on the evaluation of the existing knowledge about these pathogens, this paper gives recommendations for prevention and infection control measures as well as proposals for various target groups to tackle the threats posed by Gram-negative bacteria and prevent the spread and emergence of new antibiotic resistances.

## Zusammenfassung

In den letzten Jahren haben die durch multiresistente Gram-negative Bakterien (MRGN) verursachten Infektionen in allen Teilen der Welt dramatisch zugenommen. Der vorliegende Konsensus basiert auf Vorträgen mit sich anschließenden Diskussionen und späterer Auswertung der einschlägigen Literatur durch ein internationales Expertengremium, das von der Rudolf Schülke Stiftung, Hamburg, zu dem Meeting nach Hamburg eingeladen worden war. Im Fokus standen die Epidemiologie und die besonderen Eigenschaften Gram-negativer Bakterien, die Ausbreitung der Antibiotikaresistenz, die Reservoire, Übertragungswege und Risikofaktoren für Infektionen, die Mortalität, die Therapie und die Möglichkeiten der Prävention einschließlich der Konsequenzen des Vorkommens in der industriellen Tierhaltung. Dem One Health Ansatz folgend und basierend auf der Bewertung des Wissensstandes zu diesen Erregern werden Empfehlungen zur Prävention und Bekämpfung sowie Vorschläge für verschiedene Zielgruppen unterbreitet, um der Bedrohung durch MRGN zu begegnen, ihre Ausbreitung zu verhindern und die Entstehung neuer Antibiotikaresistenzen zu unterbinden.

## 1 Introduction

Antibiotic resistance has been referred to as *“the silent tsunami facing modern medicine”* [1]. It has been the topic of numerous international health summits and political summits. An abundance of comprehensive reports, guidelines and recommendations both on an international and on a national level have been published to tackle the threats posed by antibiotic resistance. Despite this awareness in science and politics and the more recent attention by mass media, antibiotic resistance continues to increase throughout the world. 

In this context, the rise of multidrug resistance in Gram-negative bacteria (MDR-GNB) has become a particularly serious challenge for healthcare professionals. During a two-day symposium held by Rudolf-Schülke-Stiftung, Hamburg, an expert panel of renowned infection preventionists from Germany, Austria, India and the U.S. discussed current perspectives with regard to prevention of emergence and spread of multidrug-resistant Gram-negative bacteria (MDR-GNB). The consensus report below outlines key elements of a One Health approach, taking into considerations various aspects such as special features of Gram-negative bacteria and their antibiotic resistance, the epidemiological situation worldwide, reservoirs, transmission paths, risk groups, treatment options, and effectiveness of existing prevention strategies.

## 2 Main properties of Gram-negative bacteria and multidrug resistance in Gram-negative bacteria

Gram-negative bacteria (GNB) differ from Gram-positive bacteria with respect to the structure of the cell wall. This results in differences in the penetration and retention of chemical agents. Gram-negative bacteria have what is referred to as envelope, consisting of three principal layers:

1. the outer membrane, containing the (possibly fatal) lipopolysaccharide/endotoxin,
2. the peptidoglycan cell wall with peptide chains, partially cross-linked, and
3. the cytoplasmic or inner membrane.

Gram-positive bacteria generally lack the outer membrane. The main function of the outer membrane is to serve as a permeability barrier, excluding certain drugs and antibiotics from penetrating the cell [2]. This feature is one of the main factors contributing to the *intrinsic* antibiotic resistances observed in Gram-negative bacteria.

Medically important Gram-negative bacteria include the following pathogens:

- *Acinetobacter* spp.
- *Bordetella pertussis*
- *Campylobacter* spp.
- *Enterobacteriaceae*: *Citrobacter* spp., *Enterobacter* spp., *Escherichia coli*, *Klebsiella* spp., *Salmonella* spp., *Serratia marcescens*, *Shigella* spp., *Yersinia* spp.
- *Haemophilus influenzae*
- *Helicobacter pylori*
- *Legionella pneumophila*
- *Neisseria* spp.
- *Pseudomonas aeruginosa*
- *Vibrio cholerae*

Gram-negative bacteria can *acquire* resistance to one or more important classes of antibiotics, which usually prove effective against them such as:

1. Ureidopenicillins (piperacillin)
2. Third- or fourth-generation cephalosporins (cefotaxime, ceftazidime)
3. Carbapenems (imipenem, meropenem)
4. Fluorquinolones (ciprofloxacin)
5. Polymyxins (colistin and polymyxin B)
6. Aminoglycosides (gentamicin, amikacin)
7. Glycylcycline (tigecycline)
8. Tetracyclines (doxycycline, minocycline)
9. Chloramphenicol
10. Sulphonamides (co-trimoxazole)
11. Fosfomycin

Definition of multidrug resistance varies between countries. In Germany, in the context of hospital hygiene, the term multidrug-resistant organism (MDRO) is used for Gram-negative bacteria which are resistant to three or four out of the first four antibiotic groups listed above. More specifically, **3MDRO** (*German: 3MRGN*) means multidrug-resistant Gram-negative organisms exhibiting resistance to three out of the first four antibiotic groups stated above, **4MDRO** means resistance to all four groups. It is important to note that the antibiotic groups are not considered to be equally clinically relevant. Hence, beta-lactamase resistant *Enterobacteriaceae*, which are sensitive to fluorchinolones and carbapenems, are not included herein [3].

An international panel of experts developed the following definitions: **Multidrug-resistant (MDR)** means acquired non-susceptibility to at least one agent in three or more antimicrobial categories, **extensively drug-resistant (XDR)** is defined as non-susceptibility to at least one agent in all, but two or fewer antimicrobial categories, and **pandrug-resistant** as non-susceptibility to all agents in all available antimicrobial categories [4]. When comparing and evaluating studies or guidelines and recommendations existing differences in definitions should be kept in mind.

Among these acquired resistances, “The Big Five Carbapenemases” are of particular relevance. The “Big Five” are:

1. KPC (*Klebsiella pneumoniae* carbapenemase)
2. IMP (*Imipenemase* metallo-beta-lactamase)
3. NDM (New Delhi metallo-beta-lactamase)
4. VIM (Verona integron-encoded metallo-beta-lactamase)
5. OXA (Oxacillin carbapenemases)

*Enterobacteriaceae*, primarily *Escherichia coli* and *Klebsiella pneumoniae*, are among the most frequently affected bacteria. Carbapenems are often the last line of effective treatment available for infections with MDRO *Enterobacteriaceae*. 

Until recently, the polypeptide colistin has been used as a reserve antibiotic for the treatment of critically ill patients in the event of multidrug resistance of GNB, especially in the event of resistance to carbapenem. However, the emergence of the transferrable gene mcr-1, which causes resistance to colistin, is now being reported from several countries, including China, the U.K., Denmark, the U.S. and Germany where it has been detected in intestinal bacteria in farm animals. Resistance to colistin is particularly common in isolates of *Escherichia coli* and in salmonella from poultry populations [5], [6]. In China, the gene was found in humans, including residents of long-term-care facilities, as well as in animals and foodstuffs [7], [8], [9], [10]. 

Colistin belongs to the polymyxin group of antibiotics. It binds to lipolysaccharides and phosopholipids in the outer membrane of GNB. All resistance mechanisms studied so far in this context eventually result in a reduced affinity of polymyxin to the bacterial surface [11].

## 3 Acquisition and spread of antibiotic resistance in Gram-negative bacteria

Antibiotic resistance is essentially a result of natural selection. Genetic variations in bacterial populations may carry mutations, which prove to be advantageous for their survival in the presence of antimicrobial agents. 

Antibiotic resistance can be intrinsic to specific microorganisms, which can be explained by their inherent structural or functional characteristics. Gram-negative bacteria are usually naturally insensitive to vancomycin because this antibiotic agent is not able to penetrate the outer membrane. *Klebsiella* exhibit an innate insensitivity towards ampicillin as a result of beta-lactamase production. *Pseudomonas aeruginosa* is naturally insensitive, e.g., to sulphonamides, tetracycline, chloramphenicol and trimethoprim.

Apart from innate resistance, bacteria can *acquire* resistance. Mutations in bacterial DNA can render antibiotics ineffective, conveying a survival advantage to the mutated bacterial strain. This basically means, bacteria without these advantages die or cannot reproduce in the presence of antibiotic agents, while resistant bacteria are able to proliferate with less competition. Consequently, the more antibiotics are used and disseminated, the greater the likelihood that resistance strains will emerge. 

Mutations in chromosomal genes can induce an increase in the expression of intrinsic resistance mechanisms (antibiotic-inactivating enzymes or efflux pumps) [12]. 

Resistance genes may also be acquired from other bacteria. They can be transferred between bacteria of the same species but also of another species or genus. Mechanisms of horizontal gene transfer include transduction, transformation and conjugation. Vectors carrying one or more resistance genes may be plasmids (resistance plasmid 1 is a common example in GNB), transposons (e.g. Tn5053) or integrons (e.g., Verona integron-encoded metallo-beta-lactamase producing GNB). As for GNB, in particular for *Enterobacteriacea*, there is evidence suggesting that resistance genes and associated insertion elements carried on plasmids are often found concentrated in large **multiresistance regions** (MRR) [13]. For instance, ESBL- and carbapenemase-encoding plasmids may carry resistance determinants for other antimicrobial groups, including aminoglycosides and fluorquinolones [12].

The possibility of a plasmid-mediated horizontal transmission of resistance genes between livestock and humans (e.g. via the food chain) has been observed for ESBL-genes and the colistin resistance gene mcr-1 in GNB [7], [8], [14]. The inter-species transmission of multidrug-resistant strains from humans to animals and vice versa was also suggested following findings by a veterinary diagnostic laboratory that the clonally related human B2-O25:H4-ST131 CTX-M-15-type ESBL-producing *E. coli* isolates is present in dogs and horses [15].

Yet another mechanism of emergence of antibiotic resistance has been investigated for *E. coli*. Induction of a mutagenic SOS-response by ciprofloxacin in *E. coli* caused changes of their rod-like shape into multichromosome-containing filaments. Bos et al. showed that initial resistance emerged from successful segregation of mutant chromosomes at the tips of filaments followed by budding of resistant progeny. They proposed that the first stages of emergence of resistance occur via the generation of multiple chromosomes within the filament and are achieved by mutation and possibly recombination between the chromosomes [16].

An in-vitro experiment with chemostat cultures of *Pseudomonas aeruginosa* by Feng et al. suggests that the development of resistance during patient treatment may be explained by a new acquisition of resistance rather than by gene transfer. They conclude from their studies that the risk of developing resistance may possibly be reduced by treating with antibiotics in the highest concentration the patient can tolerate for the shortest time needed to eliminate the infection [17].

One major factor believed to accelerate the spread of antibiotic resistance is excessive use of antibiotic agents, including also the use without treatment indication. The emergence of resistance has occurred following the introduction of each new class of antibiotics. A survey of van Boeckel et al. on the total antibiotic sales in selected countries from 2000 to 2010 reveals India to be the country with the highest consumption (2010: nearly 13 billion standard units, standard unit means a single dose unit, i.e. pill/capsule/or ampoule), followed by China (approx. 10 billion standard units) and the U.S. (more than 6 billion standard units, with a moderate decrease from 2000 to 2010). Substantial increases in per capita consumption of antibiotics were also reported from Australia and New Zealand. Consumption of polymyxin increased in each country apart from China [18]. 

Many studies have assumed that the broad spectrum of antibiotics used in livestock do not only cause resistance problems in pig populations, but also in public health [19], [20], [21]. Multidrug-resistant bacteria with zoonotic potential have developed in response to antibiotic use in food animals [22]. In addition, a correlation between the frequency of treatment and the occurrence of multidrug-resistant bacteria in animals has been demonstrated. The higher the treatment frequency (the average number of days each animal in the herd is treated with antibiotics), the higher the rate of resistance identified in isolates from animal products [23]. Thus, the use of antibiotics in both the health care system and in livestock production may promote dissemination of resistance genes as a direct consequence of selective pressure [24], [25], [26]. 

## 4 Surveillance and epidemiology

### 4.1 USA

In the U.S., the National Healthcare Safety Network (NHSN) collects data on hospital-associated infections (HAI). In their 2009–2010 report period (2,039 hospitals), a slight decline in MRSA was observed, while an increasing occurrence of multidrug-resistant Gram-negative bacteria (*E. coli, K. pneumonia, P. aeruginosa, A. baumanni, Enterobacter* spp.) was reported. Although generally less common as a causative organism of HAIs, both multidrug resistance and carbapenem resistance were reported in more than 60% of *Acinetobacter* spp. among most HAI types. 70%–80% of facilities reporting HAI with *Acinetobacter* spp. reported at least one multidrug-resistant strain [27]. A 2010–2012 survey (*Study for Monitoring Antimicrobial Resistance Trends*) on the resistance rates of intra-abdominal isolates from U.S. intensive care units and non-intensive care units revealed *A. baumannii* isolates from ICU patients to be most likely to exhibit resistance to more than four antibiotic classes [28]. The most significant overall increase in carbapenem resistance was observed for *Klebsiella* species (from 1.6% to 10.4%) in the NNIS/NHSN reporting [29].

As a consequence of this development, the *Multi-Site Gram-Negative Bacilli Surveillance Initiative *(MuGSI) was established as part of the *Emerging Infections Program* of CDC in 2012. It conducts active population- and laboratory-based surveillance in a defined surveillance catchment for six carbapenem-resistant organisms which include *Escherichia coli, Enterobacter cloacae, Enterobacter aerogenes, Klebsiella pneumoniae, Klebsiella oxytoca, *and* Acinetobacter baumannii*.

A report on *“Antibiotic Resistance Threats in the United States, 2013”* was published by the Centers for Disease Control and Prevention (CDC) [30]. The data show that most antibiotic-resistant infections happen in the general community, but most deaths related to antibiotic resistance occur in healthcare settings. In this report, current antibiotic resistance threats for each microorganism are divided in three threat levels (urgent, serious, concerning). For GNB (non-HAI included), the following categories apply:

- Carbapenem-resistant *Enterobacteriaceae* (CRE): Out of 140,000 healthcare-associated *Enterobacteriaceae* infections per year, more than 9,000 are caused by CRE (urgent threat level)
- *Neisseria gonorrhoea*: Of 820,000 cases per year, 30% now demonstrate resistance to at least one antibiotic (urgent threat level)
- Multidrug-resistant *Acinetobacter*: Of 12,000 healthcare-associated *Acinetobacter* infections, 7,000 are multidrug-resistant causing approx. 500 deaths per year (serious threat level)
- ESBL-producing *Enterobacteriaceae* (ESBL-E): Of 140,000 *Enterobacteriaceae* infections per year, 26,000 are drug-resistant causing 1,700 deaths (serious threat level)
- Multidrug-resistant *Pseudomonas aeruginosa*: Of 51,000 *Pseuodomonas* infections per year, 6,700 are multidrug-resistant causing 440 deaths (serious threat level)

However, it should be noted that the most urgent threat level pathogen in the U.S. is the Gram-positive bacterium *Clostridium difficile*, causing 250,000 infections with 14,000 deaths per year. *C. difficile* associated deaths are said to have increased by 400 % between 2000 and 2007 in the U.S. [30], [31]. *C. difficile* is also known to have an inherent resistance to several antibiotics. Moreover, a “hypervirulent” strain (NAP-1) has emerged potentially producing more toxins than other strains and exhibiting resistances to antibiotics such as vancomycin, fidaxomicin, and metronizdazole [31], [32].

### 4.2 India

India with a population of 1.25 billion and a GNI/capita of 5,350 USD compared to 44,540 and 53,960 respectively for Germany and the U.S. has a completely different healthcare structure and faces public health problems like limited access to improved water and/or sanitation and higher death rates from communicable diseases. Administration of antibiotics is often a more feasible option than access to safe potable water and medical advice. The total expenditure on health in India per capita is $109 compared to $6,471 in the U.S. Isolation facilities are scarce, inadequate disposal of biomedical and general waste and poor sanitation are examples for additional problems, and screening for multidrug-resistant organisms (MDRO) is not standard practice in most Indian hospitals.

Reports from various studies from hospitals in India suggest that the prevalence of ESBL-producing GNB range between 19% and 60%, and that of carbapenem-resistant GNB between 5.3% and 59% [33]. An alarming finding from a molecular characterization study of carbapenem-resistant *Enterobacteriacea* in Mumbai, West India, revealed that 18.5% (21/113) of the clinical isolates investigated possessed dual carbapenemase genes [34]. Moreover, recently a series of 24 cases of colistin-resistant *Klebsiella pneumoniae* has been reported from a new oncology center at Kolkata. The prevalence of ESBL- and carbapenemase-producers in this area was estimated to be 70% and 39%, respectively, resulting in a high first line use of meropenem and colistin in this hospital [35]. In a study from South India, ESBL-production was detected in 53% of isolates from patients with *community-acquired* bacteremia caused by *E. coli* and *Klebsiella* spp. Among those isolates, the authors also found resistance to multiple groups of antibiotics [36].

Antibiotic resistance has become a major public health concern in India. However, as of today, a mandatory national surveillance system, uninform strategies and quality control systems for sample selection and methods of susceptibility testing or mandatory antibiotic stewardship are lacking. A catalogue of strategies has been proposed, including educational and awareness programmes for community health services and the founding of an “Alliance Against Antimicrobial Resistance” [37]. Two new initiatives have been started in India in 2017. The first one is the Indian Council of Medical Research (ICMR) sponsored Antimicrobial Resistance Surveillance Network where several new Regional Centers have been established in addition to the previously existing nodal centers. Secondly, a CDC-ICMR and AIIMS (All India Institute of Medical Sciences, New Delhi) supported Capacity Building project on AMR and Health Care Associated Infection (HCAI) surveillance across the country were set up. Hopefully these two new initiatives will enable better infection control, better HCAI and antimicrobial resistance surveillance along with strengthening infrastructure for such activities across many regions of India (personal communication).

### 4.3 Europe

Despite great variations within the 30 states reporting to the EARS-Net (European Antimicrobial Resistance Surveillance Network) in 2016, important resistance trends for Gram-negative bacteria are recognizable [38]:

- Lower resistance levels reported in the northern and western compared to the southern and eastern European countries
- Significant increase (from 2012 to 2015) of resistances to third-generation cephalosporins in *K. pneumoniae* and *E. coli*, also in combination with ESBL
- Significant increase (from 2012 to 2015) of combined resistance of third-generation cephalosporins with fluoroquinolones and aminoglycosides for *E. coli* and *K. pneumoniae*
- EU/EEA population-weighted mean for carbapenem resistance 8.1% for *K. pneumoniae*, 0.1% for *E. coli* (data referring to 2015)
- Wide inter-country variations for carbapenem resistance in *K. pneumoniae* between 0 and 61.9%
- High percentages of combined resistance to fluorquinolones, third-generation cephalosporins and aminoglycosides often associated with reported high percentage of carbapenem resistance (for *K. pneumoniae*)
- Significant increases for carbapenem resistance and resistance to multiple antimicrobial groups also in *P. aeruginosa* and *Acinetobacter* spp.
- Carbapenem resistance for *E. coli* remained stable
- Highest levels of resistance reported for *Acinetobacter* spp. (with carbapenem resistance reaching over 80% in some countries in the south and southeastern parts of Europe and in the Baltic States)
- Isolates (e.g. of *K. pneumoniae*) with polymyxin resistance were reported by some of the countries included in EARS, showing that resistance to polymyxin is significantly higher in carbapenem-resistant than in carbapenem-susceptible isolates.

The editors of the report point out that data on polymyxin susceptibility from EARS-Net are to be interpreted with caution due to variations in methodology and potential selective testing. The joint CLSI-EUCAST Polymixin Breakpoints Working Group has published a method to determine colistin minimial inhibitory concentration distribution in 2016 [39].

As for *C. difficile*, a surveillance protocol was updated in November 2015 to improve standardization of testing and the quality of data reports [40]. It is estimated that approximately 9% of all hospital-associated infections are caused by *C. difficile* [40].

### 4.4 Germany

In Germany, the national surveillance system KISS (abbreviation for the German word for hospital infection surveillance system) collects data of nosocomial infections from certain hospital risk areas (e.g., intensive care) and high-risk patient groups (e.g., neonates). A KISS-module for the surveillance of colonized or infected ICU patients with selected MDRO, and of all hospitalized patients with MRSA and *C. difficile* associated diarrhea (CDAD) in intensive care wards also exists. The German Antimicrobial Resistance Surveillance System (ARS) is cooperating partner of the European Antimicrobial Resistance Surveillance Network (EARS-Net). In addition, a surveillance database for antibiotic consumption for hospitals has been established (AVS).

Evaluation of KISS data show that while MRSA remains stable or is slightly decreasing, other MDROs among ICU patients are becoming more common, and hospitalized patients are twice as likely to acquire CDAD as they are to acquire MRSA [41]. According to the point prevalence study in 2011, which was carried out according to the ECDC protocol, the most common pathogens found to cause nosocomial infections in Germany were *E. coli* (18%), Enterococci (*E. faecalis* and *E. faecium*, 13.2%), *S. aureus* (13.1 %), and *C. difficile* (8.1%). The five most commonly used classes of antibiotics were second-generation cephalosporins, followed by fluorquinolones, penicillins with beta-lactamase inhibitors, third-generation cephalosporins, and carbapenems. A substantial amount of *C. difficile* infections was noted, thus confirming the KISS data [42]. Maechler et al. report that the acquisition rate of carbapenem-resistant organisms (CRO) in intensive care units was more than double the rate of other MDRO under surveillance by MDRO-KISS. CRO prevalence was 0.29 per 100 patients [43]. As a result of the increase in carbapenem resistance, the German Health Ministry has released an ordinance for mandatory notification of laboratory-confirmed colonization with carbapenem-resistant organisms as of May 2016 [44].

Apart from data extracted from hospital surveillance systems, the prevalence of antibiotic resistance in the community is an important aspect for screening, infection control and treatment regimens. In a study with 3,344 participants from southern Germany, 6.3% faecal samples collected between October 2002 to November 2012 were found to be positive for ESBL-producing *E. coli* [45], suggesting that, in Germany, too, the community has to be taken into consideration as a reservoir for antibiotic-resistant organisms and travelling abroad also contributes to colonization with these bacteria.

### 4.5 Common trends

Although there are shortcomings in the report and surveillance systems throughout the world and the reliability and comparability of data is limited, there are many patterns which are evident and which call for immediate action. For example, carbapenem resistance is likely to rise as a result of antibiotic resistance to other antibiotic groups (such as 3^rd^ generation cephalosporins) and an increased use of reserve antibiotics. Similarly, colistin resistance is thought to be associated with the rise in CRO (carbapenem resistant organisms) and increased usage of colistin as a reserve antibiotic. 

Besides GNB, *C. difficile* infections, including those with drug-resistant highly virulent strains, are on the rise and need continuous monitoring. The acquisition and spread of antibiotic resistance is a rapid, dynamic, global process which is neither restricted to a specific bacterium, to specific geographical areas or environments nor to humans or animals, nor to the healthcare system. On the other hand, the fact that methicillin-resistant *S. aureus* is actually receding or being contained in some places shows that it is possible to reverse the trend by adequate policies such as systematic risk-adapted screening, standard and contact infection control precautions and antibiotic stewardship.

## 5 Methods for MDRO detection

Multidrug-resistant organisms (MDRO) may be detected directly from clinical samples (e.g. stool, rectal, swab, throat swab, wound swab, skin swab, etc.) by either culture-based phenotypic methods or PCR-based genotypic methods. Culture-based methods are generally time consuming (results taking 2–5 days), labour-intensive and may suffer from problems of relatively low analytical sensitivity. However, culture-based methods are relatively inexpensive, may be performed in laboratories without automation or advanced technology. Examples are the modified Hodge test (MHT) and the Carba NP Test [46], [47]. Genotypic methods such as multiplex and singleplex PCR amplification and pulsed-field gel electrophoresis (PFGE) are not widely available in hospital laboratories around the world and are more expensive. PCR-based methods provide more rapid results (within 1–2 days) and have a high degree of analytical sensitivity because of their ability to detect low numbers. However, unlike culture-based methods, they are not able to give detailed antibiotic susceptibility test results (except for the gene of interest) and may suffer from the problem of specificity [48], [49], [50]. 

Examples for recent developments are the RAPIDEC^®^ CARBA NP assay for rapid detection of carbapenemases in *Enterobacteriacea*, *P. aeruginosa* and *Acinetobacter* spp., an immunochromatographic assay (the OXA-48 K-SeT assay) to detect OXA-48-like carbapenemase-producing *Enterobacteriaceae* from culture colonies, as well as a highly discriminatory universal blaSHV, blaTEM and blaCTX-M subtyping assay of clinically important ESBL genes, based on PCR and Sanger sequencing [51], [52], [53], [54]. 

In addition, underestimation of the presence of multidrug-resistant pathogens may occur as GNB have the capacity to survive in the viable-but-not-culturable state (VBNC-state) as well as in biofilms, making detection by conventional methods difficult [55]. For example, multidrug-resistant *A. baumannii* has been shown to form biofilms on the surfaces of respiratory epithelial surfaces and also in the environment [56], [57].

As each bacterium and the respective resistance mechanism is associated with different epidemiological risks and new mechanisms are being detected with new diagnostic methods, it becomes apparent that a holistic and proactive approach to tackle antibiotic resistance is imperative. 

## 6 Habitats and reservoirs of (antibiotic-resistant) Gram-negative bacteria

### 6.1 Natural habitats and reservoirs

Natural habitats and reservoirs of GNB are, depending on the species, soil, water, sewage, plants (fruit, vegetables, herbs), dairy products, raw meat as well as the gastrointestinal tract of humans and animals, the skin and the upper respiratory system (cf. Table 1 (Tab. 1)). 

### 6.2 Reservoirs of Gram-negative bacteria in healthcare facilities

Investigations of outbreaks with MDR Gram-negative bacteria have revealed a number of reservoirs in hospitals. These are, for example:

- Colonized/infected patients
- Biofilms, in humidifiers of incubators in neonatal wards and other devices which are likely to form biofilms (cf. [55] (Table 1 (Tab. 1)); [58])
- Sinks/hand washing basins, faucets, drains, sink traps [59]
- Standard toilets with a rim [60]
- Sewage/drainage system [61], [62], [63]
- All surfaces in hospitals such as beds/bedside surfaces, fabrics (linen, pillows, privacy curtains) [55], [64]
- Computer keyboards and faucet handles [65]
- Duodenoscopes/endoscopes [66]
- Hospital foodstuff [67]

Most GNB thrive in a moist, humid environment. Consequently, typical reservoirs include shower heads, bars of soap, liquid soap, artificial fingernails, pools/hot tubs/water fountains, dialysis tubing, infusion pumps, respiratory equipment and cleaning mops. However, according to a systematic review of the persistence of nosocomial pathogens on inanimate surfaces, certain Gram-negative species are also able to survive on inanimate, dry surfaces, even for months. Prominent examples are *Acinetobacter* spp., and *Klebsiella* spp. [68], [69]. On the other hand, according to a recent study by Weber et al. [70], carbapenemase-resistant *Enterobacteriaceae* on various actively inoculated surfaces in CRE-patient rooms showed less than 15% survival within 24 hours and only infrequent (8.4%) and low levels of contamination (5.1 cfu/120 cm^2^). Further studies are necessary before concluding from these findings that the risk of CRE-transmission from environmental surfaces is relatively low (cf. comment by A. Voss [71] on [68], [70]). 

Outbreak investigations can contribute to the identification of transmission paths within medical facilities. Apart from direct person-to-person transmission involving (the hands of) colonised or infected patients, visitors and staff, transmission via all kinds of surfaces as well as aerosols, both dust and droplets, must be taken into consideration. The sewage system and horizontal drainage systems as reservoirs have proven to be associated with various possibilities of transmission paths. One recently discovered path were clogged pipes, which caused backwater, which can, in turn, contaminate sinks. When spirales (drain snakes) were used to unclog drains and are thereafter used in different areas of a hospital, the pathogens can be transferred via the spirales [72]. Cleaning processes with aerosolization such as high pressure cleaning with high-pressure water/steam bear the risk of contaminating food or surfaces. All ecological niches for Gram-negative bacteria such as sink drains, traps and toilet brims can become a risk when surrounding surfaces (e.g. of counters used for preparing infusions) or hands become contaminated by aerosol/droplet formation. It is important to note that plasmids carrying resistance genes can be transmitted across species barriers as was the case in the multi-species outbreak described above [72].

### 6.3 Reservoirs of Gram-negative bacteria in livestock

Livestock, especially pigs, cattle and poultry, as well as pets and sheeps are a well-known reservoir for MDR-GNB (e.g. [7], [15], [73], [74], [75], [76]). The primary focus here is on ESBL-producing *Enterobacteriaceae* (ESBL-E). Hot spots for transmission in the pig production chain are the stable air as well as stressful crowding situations, e.g. in humid, warm waiting areas before the abattoirs, whereas carcasses in cold store do not constitute a major problem. While it has been clearly established that farmers are at a higher risk of acquiring MRSA than the general population – primarily as a result of transmission by air/contaminated dust – the risk of colonization with ESBL-E has not been fully elucidated [77]. However, studies do exist suggesting that a direct transfer from livestock to humans is possible [78]. 

Transmission of MDRO via food products and water is also possible. Numerous publications have demonstrated that drinking water, meat products or milk can be contaminated with MDR-GNB and constitute a potential vehicle for pathogen transmission [7], [21], [79], [80], [81], [82], [83]. 

Moreover, with the projected change in farming systems in middle-income countries towards large-scale intensive farming, routine use of antibiotics in subtherapeutic doses, which are likely to accelerate resistance, is bound to increase. According to van Boeckel et al., global consumption of antimicrobials in livestock is estimated to rise by 67% from 2010 to 2030 [84].

## 7 Mortality from antibiotic-resistant Gram-negative bacteria

Infections caused by MDR-GNB include urinary tract infections, blood stream infections (sepsis), pneumonia, meningitis, diarrhea, gonorrhea, otitis, ocular infections including endophthalmitis, device-related infections (e.g. related to central vascular catheter placement) and wound or intraabdominal infections. 

Between October 2014 to May 2015, 40 carbapenem resistant blood stream infections were observed in patients admitted to Tata Medical Center, Kolkata, India. These were due to *E. coli, K. pneumoniae, P. aeruginosa* and *A. baumannii* in 12, 15, 8 and 10 patients respectively. The median age of the patients in this group was 48 years (range: 9.5 to 84.3 years). Twenty-one patients had haematologic malignancy and 19 had solid organ cancers. The 30-day all-cause mortality noted because of these four multi-drug resistant bacteria (*E. coli, K. pneumoniae, P. aeruginosa* and *A. baumannii*) was 0%, 40%, 50% and 60%, respectively. The median time to mortality was 3 days after a positive blood culture with carbapenem resistant Gram-negative bacteria (range 1–30 days). Stool surveillance cultures done in patients admitted to the hospital during the same period showed that 85% of the patients were colonised with one or more MDROs (n=156). Almost all *E. coli* and *Klebsiella* isolates in stool were ESBL-producers. 20% of the *E. coli* and 45% of *Klebsiella* isolates in stool were carbapenem-resistant. Similarly, 36% of throat swab surveillance cultures showed one or more MDROs (n=55). 40% of the *Klebsiella* isolates from throat swabs were carbapenem-resistant [85].

Data from WHO [86] confirm that the risk for bacterium-attributable death from antibiotic resistant microorganisms is significantly greater – twice that of patients with non-resistant bacteria (e.g. this applies to *E. coli* which are resistant to 3^rd^ gen. cephalosporins, *K. pneumoniae* with resistance to carbapenems). In their metaanalysis of nine studies published up to April 2012 on mortality following carbapenem-resistant *Enterobacteriaceae* infections, the authors calculated that 26–44% of deaths in 7 studies were attributable to carbapenem resistance [87]. They compared all-cause deaths of patients with carbapenem-resistant infections with those with carbapenem-susceptible infections. On the other hand, an analysis of 24 studies for acquiring infections caused by MDR-GNB in ICU published in 2016 cannot confirm a direct association between infections due to Gram-negative MDR bacteria and mortality in ICU patients, which supports the need for further studies [88].

One of the main reasons for high mortality due to MDR-GNB infection is a late onset of appropriate initial antibiotic therapy. Later administration of a suitable antibiotic was found to be associated with an adverse outcome, particularly in patients with a high-risk source such as lungs, peritoneum or unknown origin of bacteraemia caused by *E. coli, K. pneumoniae, Enterobacter* spp., and *P. aeruginosa*. The adequately treated patient group had a 27.4% mortality rate compared to 38.4% for the inadequately treated group. These findings are underlined by observations that the median time from detection of the positive blood culture to the time of death can be very short (1–5 day for *P. aeruginosa* and 1–30 days for *Acinetobacter* in the surveillance report from Tata Medical Center, India, see paragraph above [85]). Thus, for the calculated antibiotic therapy, the immediate initiation of treatment (within the first hour after diagnosis) is crucial for the survival of the patient. In the first 6 hours, the mortality risk of the antibiotic untreated septic shock was reported to increase hourly by 7.6% [89]. 

In everyday practice, appropriate antibiotic therapy is not always readily available on site, even if effective antibiotics exist such as colistin for CR *Klebsiella*-induced pneumonia. In a study from Greece the vital role of combination therapy in the management of MDRO bacteraemia was explored. It was found that out of the 205 patients with blood stream infections caused by carbapenemase-producing *K. pneumoniae*, the all-cause 28-day mortality was 40%. A significantly higher mortality rate was observed in patients treated with monotherapy than in those treated with combination therapy (44.4% versus 27.2%; P=0.018). The lowest mortality rate (19.3%) was observed in patients treated with carbapenem-containing combinations. Combination therapy was strongly associated with survival (HR of death for monotherapy versus combination, 2.08; 95% CI, 1.23 to 3.51; P=0.006), mostly due to the effectiveness of the carbapenem-containing regimens [90].

## 8 Risk factors for acquiring infections caused by MDR-GNB

### 8.1 Hospital patients 

The following risk factors associated with healthcare-associated infections caused by antimicrobial resistant versus susceptible *A. baumannii* were identified for U.S. hospitals [91]:

- Antibiotic use prior to infection
- Length of stay prior to infection
- Hospital A vs. B
- Respiratory infection
- Active duty in Iraq (“Iraqibacter of soldiers”) [92], [93]

Similarly, in a systematic review of observational and experimental studies (published up to October 2015) with regard to the prevalence of antibiotic resistance in urinary tract infections caused by *E. coli* in children and young people aged 0–17, the authors infer that children who had previously received antibiotics in primary care were more likely to exhibit resistance to antibiotics persisting for up to six months after treatment [94]. 

Previous carriage of resistant organisms appears to be another risk factor. In a case-control study including 276 CRE carriers in whom CRE carriage presumably ended, following at least 2 negative screening samples on separate days, Bart et al. found 36 (13%) to show recurrence within one year after presumed eradication [95]. The recurrence rate was 25% when the carrier status was presumed to have been eradicated 6 months after the last known CRE-positive sample, compared with 7.5% if presumed to be eradicated after 1 year. The authors conclude that the CRE carrier status should be maintained for at least 1 year following the last positive sample and they advise screening of all prior CRE carriers independent of their current carriage status. Duration of carriage is also extended in case of multiple hospitalization within one year [96]. 

With regard to specific hospital wards, neonates and immunocompromised patients from intensive care units, oncology and transplantation wards are at special risk. A large number of studies confirm risk factors such as recent organ or stem-cell transplantion, receipt of mechanical ventilation, extended hospital stay, previous treatment with cephalosporins and carbapenems [97], [98], [99]. Qureshi et al. recently reported 20 patients at Pittsburgh Med Center known to be infected or colonized with colistin-resistant* A. baumannii*. It was found mainly among patients who had received colistin methansulfonate (i.v. or inhaled) for treatment of carbapenem-resistant, colistin-susceptible *A. baumannii* infection (predominantly ventilator-associated pneumonia) prior to identification of colistin resistance. A 30-day all-cause mortality rate of 30% was recorded. Lipid A modification by the addition of phosphoethanolamine was responsible for colistin resistance [100]. 

In January of 2016, the German Robert Koch Institute published a statement on the question of screening of refugees and asylum seekers for MDRO which is kept updated [101]. Few existing studies do suggest that the proportion of asylum seeking people, including unaccompanied minors, which are colonised with multidrug-resistant ESBL-forming Gram-negative bacteria is significantly higher than that of the general German population [101], [102], [103]. The available national screening guidelines have not been changed. They recommend to screen patients upon admission to hospital for MRSA and carbapenem-resistant organisms in case they are from regions with a high prevalence of MDRO or in case they had contact to the healthcare system outside of Germany or if their clinical history is unclear. In the most recent study published in Germany, 9.8% of the refugees were colonized with MRSA, and 23.3% with resistant Gram-negative bacteria [104]. Therefore, the authors recommend screening and special infection control measures in hospitals when refugees are admitted to hospitals, in order to ensure best medical practice and safety for all hospital patients regardless of their country of origin.

### 8.2 Nursing homes and long-term care residents

Age >60 years is independently associated with MDR-GNB [105]. New acquisition of MDR-GNB in nursing homes and long-term care facilities within a relatively short period of time is common in the United States and may reach up to 50%, depending on the species [106], [107]. Prevalence of MDR-GNB is now being reported to exceed that of vancomycin-resistant Enterococci and MRSA in the U.S. [108]. Molecular typing suggests that person-to-person transmission within common areas in LTCF (long-term-care facilities) is one important path of transmission. 

The following risk factors have been identified for various MDR-GNB among residents in nursing homes and/or long-term care facilities (cf. [107], [108], [109], [110], [111], [112]): indwelling devices (urinary catheters and/or feeding tubes), faecal incontinence, functional disability, diabetes mellitus, previous antibiotic exposure, travelling to high prevalence countries and antimicrobial use during travels, advanced dementia. In a prospective cohort study including 22 nursing homes in the greater Boston area (U.S.A.), advanced dementia was associated with a spread of MDR-GNB within a nursing home but also between nursing homes. Genetically related MDR-GNB strains were detected in 18 of the 22 nursing homes. Residents with advanced dementia should therefore be regarded as a high-risk group. 

### 8.3 Neurological rehabilitation clinics and intensive in-home care

Neurological rehabilitation clinics also show high prevalence rates of MDRO, including GNB. Risk factors include direct transfer from acute care hospitals, previous antimicrobial treatment during the past 3 months and wounds [113]. 

Other non-acute settings outside the hospital which are often neglected as risk factors include ambulatory care-givers [113], intensive in-home care or structured residential services (group care settings) [114], [115].

### 8.4 Leisure and business travel, medical tourism

According to a systematic assessment of 11 studies (published up to August 2015) conducted by Hassing et al., international travel is considered to be an important risk factor for the carriage of multidrug-resistant *Enterobacteriaceae*, with prevalences of >20% [116]. In particular, the risk is increased for persons travelling to (southern) Asia and for persons with travel-related diarrhea and antibiotic use. In the southeastern Asian countries Thailand, Singapore, Malaysia, Vietnam, Indonesia, Philippines, Laos, Cambodia, Myanmar, Brunei 39.4% of 436 isolates (*E. coli, K. pneumoniae, K. oxytoca* and *P. mirabilis*) were positive for ESBL production. The prevalence of carbapenem-resistant (CR) *A. baumannii* varied between 76 and 90%, of CR *P. aeruginosa* between 23 and 47% [117]. Similarly, Kuenzli et al. found high colonization rates of ESBL-producing *E. coli* (ESBL-E) in Swiss travellers to South Asia [118]. In a prospective cohort study with 275 German volunteers travelling to 53 different countries stool samples and travel-associated risk factors such as type of travel, nutritional habits, occurrence of gastroenteritis were investigated before and after the journey [119]. Pre-travel analysis demonstrated a ESBL-E colonization rate of 6.8%. Thirty point four percent of the previously uncolonized subjects were colonized with ESBL-producing *E. coli*, and 8.6% were carriers of ESBL-producing *Klebsiella pneumoniae* upon returning to Germany, especially from those travelling to India and to South East Asia. The authors recommend active surveillance and contact isolation upon admission to healthcare facilities for patients having travelled to India and South East Asia in the previous 6 months [119]. Colonization of travellers with MDR-GNB appears to be transient and disappears after 3–6 months [120]. 

Apart from holiday and business travel, medical tourism and wellness tourism may be potential risk factors. According to the Medical Tourism Facts and Figures 2015 Report (International Medical Tourism) 6 million people travel for medical treatment from one country to another [121]. A comprehensive survey issued by OECD criticizes the *“grave lack of systematic data concerning health services trade, both overall and at a disaggregated level in terms of individual modes of delivery, and of specific countries. This is both in terms of the trade itself, as well as its implications”*. The report explicitly mentions infection and cross-border spread of antimicrobial resistance and dangerous pathogens as a risk associated with the treatment processes [122]. 

Based on the important findings concerning risk assessment of MDR-GNB described above, the subsequent chapters will pinpoint important treatment and prevention principles.

## 9 Principal treatment options

The current paradox is that while antibiotic resistance and associated morbidity and mortality are increasing, research for new antibiotics and novel mechanisms of action has been decreasing. High costs for the development and a poor return of investments are considered to be the main reasons for this situation. Only a few new agents are presently under clinical trial, among them the non-β-lactam β-lactamase inhibitor avibactam and plazomicin, a novel aminoglycoside [123], [124], [125]. 

### 9.1 Antibiotic stewardship

With respect to treatment options of infections caused by MDR-GNB suitable and regionally adopted antibiotic treatment regimens are to be laid down for each medical facility and expert decisions have to be made for each individual patient. For example, tigecycline and doripenem have been successfully tried for treating infections caused by multidrug-resistant *Acinetobacter* spp. As a rule, using the highest concentration of an antibiotic the patient can tolerate for the shortest time needed to eliminate the infection is recommended [126], [127], and combination treatment may offer an advantage over monotherapy in critically ill patients with severe infections caused by carbapenemase-producing *Klebsiella* spp. [128]. On the other hand, combination regimens are often associated with more severe side effects [129]. 

### 9.2 Decolonization

Topical (skin) decolonization of MDR-GNB has proven difficult or ineffective. A recent review of various decolonization agents indicated that chlorhexidine gluconate and sodium hypochlorite show the strongest evidence for activity against Gram-negative organisms [130]. Use of triclosan, hexachlorophene and povidone-iodine is not recommended at this time. 

Selective digestive and oropharyngeal decontamination as a measure to prevent surgical site infection may have a positive effect [131], but more studies are needed to verify the benefits and to investigate a possible selection for resistance among Gram-negative bacteria.

### 9.3 Antisepsis

Other novel treatment approaches such as the use of antiseptics, probiotics, and bacteriophages are presently being studied. The topical application of systemic antibiotics for skin, mucous membrane and wound infections is obsolete with only one exception: The same antibiotic is given orally or parenterally in case of metastatic infection [132], [133]. Antiseptics are more effective than antibiotics in vitro [134], [135], equally effective in vivo [136], [137] and, administered topically, as compatible as antibiotic eye drops [138]. In contrast to antiseptics with a microbiostatic specific mode of action (chlorhexidine, triclosane, QAC, silver ions [cf. [138], [139], [140]]), microbicidal antiseptics are without risk of development of microbial resistance [141], [142], [143], [144].

## 10 Prevention strategies

### 10.1 Control of resistance emergence and dissemination

In the 2014 draft of the *Review on Antimicrobial Resistance* issued by the U.K. government [145] the authors begin with the optimistic statement on the chances to bring the growing threat of antibiotic resistance under control: “We believe that this crisis can be avoided. The cost of taking action can be small if we take the right steps soon. And the benefits will be large and long-lasting especially for emerging economies […].” However, if antimicrobial resistance is left untackled, they continue, an estimated 10 million deaths per year attributable to AMR every year by 2050 will be the consequence. In the final document published in 2016, O’Neill, chair of the review, reflects “Indeed, even at the current rates, it is fair to assume that over one million people will have died from AMR since I started this Review in the summer of 2014. This is truly shocking.” [146].

In order to prevent further spread, it is crucial to eliminate sources for resistance development and reservoirs for multidrug-resistant bacteria in the environment. Human, agricultural, aqua farming, hospital and industrial waste and waste water are a path for antimicrobials as well as resistance genes and gene pools originating from a vast bacterial spectrum via waste water treatment plants, waste water and sludge/manure, thus contaminating drinking water, surface water/groundwater and agricultural soil. Standardized, reliable testing of environmental samples must be employed to detect the contents of resistant bacterial strains and antimicrobial residue/agents in surface water, agricultural soil and drinking water in order to adopt necessary control measures. 

The extent to which certain sub-lethal concentrations of antimicrobial agents may induce resistance and facilitate horizontal gene transfer of resistance genes has not yet been fully eludicated [147], [148]. 

One major prevention strategy is to curtail production, prescription and consumption of antibiotics both in human and in veterinary medicine. Education of the general population, of healthcare personnel, veterinarians and pharmacists about means of prevention and proper treatment of infections is also indispensable for this process. Obligatory antibiotic stewardship programs in human and in veterinary medicine, in dentistry as well as in animal breeding should be enforced throughout the world. 

An equally important critical control point is the approach to eliminate the need to employ antibiotics by offering access to clean, affordable water and sanitation to all people, by implementing infection control precautions in medical facilities and everyday life, by promoting vaccination, and by introducing animal breeding and food-production processes which render the use of antibiotics unnecessary. Standardized surveillance and mandatory notification of infections caused by multidrug-resistant organisms supply crucial data for continual risk assessment and risk management decisions. 

A plethora of guidelines, recommendations and strategic action plans, frameworks for action, including manuals for developing national action plans with support tools already exist on a national and international level, on the prudent use of antibiotics, on combating emergence and dissemination of antimicrobial resistance, on One Health approaches to tackle antibiotic resistance, on antibiotic use and resistance in food animals (cf. e.g. websites of WHO, ECDC, CDC, CDDEP (Center for Disease Dynamics, Economics & Policy), national public health services and health ministries). Policy makers must be called upon to ensure that these strategies are implemented and their implementation is strictly adhered to. 

### 10.2 Reservoir- and transmission-based prevention strategies in healthcare and long-term care facilities

Prevention strategies in healthcare include **reservoir-based and transmission-based measures**. They may be classified in different levels of evidence (e.g. strong and conditional or classes I through IV, as in the U.K. and German national recommendations, respectively [3], [149]. Even though Gram-negative bacteria require special attention, they are always part of a bundle of measures directed to all potential pathogens in a particular environment.

The following strategies are generally recommended:

- Implementing a mandatory **antibiotic stewardship regimen**
- **Surveillance**: Recording, reporting and evaluating multidrug resistance
- **Patient history and risk-based screening**: Assessing travel history (business, leisure, refugees), screening of risk patients, contact precautions
- **Assessing medical history/hospital stay** within the last 12 months and screening of risk patients, contact precautions
- **Training and education** of personnel responsible for screening and contact precautions
- Principle of **rapid diagnosis**, quick transmission of information, quick treatment: Alert or flagging system (flagging and electronic recording of MDRO carriers in patient charts/patient database), appropriate information of caregivers, patients, visitors and all hospital personnel
- Monitoring and **reinforcing infection control standard precautions**
- Additional **contact precautions** for patients known to be or to have been colonized or infected with MDR-GNB
- **Safe decontamination practices and cleaning protocols**: e.g., avoiding aerosol formation (foam, sprays), avoiding bacterial dissemination by inadequate disinfection techniques and materials, avoiding incorrect dosage
- **Antiseptic prevention and therapy of localized infections**
- **Discarding secretions/body fluids** in designated areas and cleaning sinks (not in hand wash sinks)
- **Patient education**: toilet use, emptying of urinary bags, etc.
- **Safe disposal of (hospital) waste** including safe standard procedures for removing blockage/clogging.

Compliance is a major issue often discussed in this context, including one special aspect: training and education of patients and their families along with the staff may increase compliance with and effectiveness of existing guidelines. As a consequence, patients may be able to participate in social and family-centred care programs which would otherwise not be an option [150].

The following measures are subject to debate and/or vary according to availability of resources:

- Active screening: who, which microorganisms, how, with which consequences?
- Antiseptic skin wash?
- Single room and/or cohort isolation [151], [152], [153]?
- Environmental sampling: when, where and how?
- Monitoring disinfectant effectiveness: how often and how?

#### 10.2.1 Reservoir-based prevention

As mentioned earlier (Chapter 6), potential reservoirs for MDR-GNB such as the plumbing system, sanitary facilities as well as medical instruments, e.g. complex endoscopes, are often difficult to decontaminate. Therefore, it is an interesting topic to consider modifications in technical or/and architectural design:

- Hygienically intelligent/optimized sanitary facilities like rimless toilets, appropriate sink and faucet design (sloped angles to minimize splashing, offset drain, sealed overflow, faucet spout for easy maintenance). New self-disinfecting sink drains where shown to reduce the *P. aeruginosa* bioburden in a neonatal intensive care unit [154].
- Optimized workflows and space for 2 beds per room as a standard, sinks with appropriate design (see above), sufficient space for personal belongings.
- No cabinets or storage areas beneath sinks.
- Point-of-use water filters in facilities for immunocompromised patients.
- Measures to prevent biofilm formation (adjustment of water flow rate and water pressure).
- Materials/medical devices/surfaces which are easy to clean and disinfect and which prevent biofilm formation (e.g. specific (coated) materials for urinary catheters).
- Maintaining a good quality of water supply in hospital and community settings (for example through adequate and appropriate levels of chlorination) and checking the water quality microbiologically by sensitive techniques [155].

#### 10.2.2 Example for transmission-based prevention strategies: Disinfection of surfaces

Effective disinfection is one of the most important parts of the multi-barrier approach to prevent dissemination of MDR-GNB [156], [157]. This is not only applicable to areas of medical care of humans, but also to veterinary medicine and, for example, to livestock breeding. Schmidthausen et al. demonstrated that a comprehensive cleaning and disinfection regimen as part of an intervention bundle in the stables of a pig farm was able to effectively eradicate MRSA and β-lactamase producing *Enterobacteriaceae* [158]. 

Frequently used biocides contain quaternary ammonium compounds, alcohols, aldehydes, peroxides, chlorine compounds, amphoteric substances. In a comparative evaluation of the efficacy of surface disinfectant cleaners composed of different active agents against multidrug-resistant clinical isolates of GNB, Reichel et al. confirmed that the tested surface disinfectants exhibited sufficient efficacy when tested according to the method described in the standard EN 13727:2012 under dirty conditions. However, in their conclusion the authors do state that individual clinical isolates (e.g. one 4MRGN *P. aeruginosa* isolate) might exhibit reduced susceptibility to selected biocidal agents (e.g. aldehyde-containing surface-active substances) [159]. 

Also, quaternary ammonium compounds have been found to be less effective against *S. marcescens* than other active agents: Investigations to clarify an outbreak with *S. marcescens* in a German hospital showed that disinfection with a product containing 3 quaternary ammonium compounds was ineffective against *S. marcescens* clinical isolates which were detected on the lid and the bottom of a disinfectant wipe container. The removal of the first wipe with the lid open was thought to have caused contamination. When products containing peroxides were used, disinfection was successful (paper presented by Goroncy-Bermes at the RSS Symposium November 2015 [160]). Recent research has indicated that bacterial genes (qac genes) may encode efflux pumps capable of expelling quaternary ammonium compound structures from bacterial cells (MRSA), rendering these compounds less effective [161]. A high prevalence of qacE resistance genes was found among clinical isolates of MDR *Acinetobacter baumannii* in a Malaysian tertiary care hospital [162]. Silveira et al. report a hospital sewage ST17 *Enterococcus faecium* with a transferable Inc18-like plasmid carrying genes for resistance to antibiotics and to quaternary ammonium compounds [163]. 

Still, as regards the correlation between non-susceptibility to surface disinfectants and to antibiotics, research so far suggests that in general there is no risk of selection for antibiotic resistance provided disinfectants are used in the correct dosage [164], [165], except for Triclosan and quaternary ammonium compounds (QAC) which are among the active agents suspected to trigger resistance, especially when used in sublethal doses (cf. [166], [167], [168]).

These findings show that continuous research and development in disinfection is essential. Legal challenges such as the Biocidal Products Regulations, which will lead to a reduction in the active agents available for disinfection in the medical field, complex approval processes for marketing biocides and incurring costs impede investments in innovative research by industry. Rather than taking risk/benefit assessment as the basis for classification of substances, the hazard of a substance is evaluated independent from its route of exposure and its health benefits. User acceptance of effective substances with a long list of hazard labels will be low and substances for targeted, niche application for professional use will become scarce. Thus, there is growing concern that the availability of effective biocides in future cannot be ensured (paper presented by Oltmanns at the RSS symposium November 2015 [169]).

Apart from the availability of suitable active agents, training and education of cleaning staff, disinfection protocols which are easy to implement and disinfection techniques which are easy to perform are an essential contribution to safe and effective disinfection.

#### 10.2.3 Prevention strategies in agriculture: Example of pig stables in Germany 

Experimental studies on the decontamination of pig stables [170] were based on knowledge that hygiene and sanitation measures are a compelling necessity to combat endemic hospitalism (autochthonous risk) in modern livestock production [171], [172], [173]. This means preventing and inhibiting the spread of facultative pathogenic bacteria that occur in animal husbandry and in hospitals and healthcare systems (hospitalism germs) [173]. In this context there is an increasing demand for the further development of new concepts and procedures to control or even eradicate drug-resistant bacteria and, thus, interrupt infection chains. Single treatment of animals for purposes of decolonization or therapy, similar to practices in human medicine, requires too much effort and fails entirely if high numbers of animals are affected within a large herd [173], [174], [175]. Moreover, not only do animals present potential reservoirs or vectors for transferring resistance genes and resistant bacteria, but farmers and farm workers, the stable environment including surfaces, installations, and many more factors should also be considered for their transmission potential [176], [177], [178]. Generally, farm management systems should include specific hygiene and sanitation measures to interrupt infection chains [173], [179], [180]. The twelve measures of prophylaxis and therapy of Mayr [181] coincide, to a large extent.

##### Sanitation of the stable environment 

Although all of the recommendations given below are based on the infrastructure and regulations in Germany, they may give some ideas and trigger developments for sanitation measures in agriculture in other countries.

- Construct a closed-off quarantine stable and sick stable in which potentially introduced diseases can be controlled or treated in order to prevent an outbreak [173], [182], [183].
- Uninstall all technical installations and supply lines, including water/feed and ventilation systems [179], [180].
- Remove damaged floors, separations, and all materials (wood, etc.) that could provide potential reservoirs for pathogens and replace with easily cleanable and smooth materials [174].
- Exchange technical equipment (driving boards, etc.) within individual stables and compartments [184].
- Disinfest manure according to stipulations of the waste removal law [173], [185].

##### Elimination and reduction of pathogenic bacteria

- Clean using a high-pressure cleaner and water.
- Foam and repeat cleansing with water.
- Dry all objects and surfaces to be disinfected [173].
- Apply disinfecting agents with bactericidal, virucidal, fungicidal, and antiparasitic properties to all surfaces, in wet form, in sufficient concentration [186] and nebulize hot steam onto all hard-to-reach spots (for example, main ventilation shafts, etc.). This final disinfection serves to eliminate MRSA and ESBL *E. coli*, as well as other bacterial groups [187].
- Allow sufficient drying time and “stable rest” before resuming stabling of any animal [173].

##### Enhancement of the individual defense mechanisms and control of the resistance status

- Limit the purchase of new pigs to those that are accompanied with health certificates from the supply farms [181].
- Specifically vaccinate with a focus on the viral respiratory diseases that have a high prevalence in the applicable region [181].
- Certification of a negative MRSA and ESBL *E. coli* status by appropriate monitoring [188].

It has to be emphasized that combating antibiotic resistance has to based on a multi-faceted approach comprising the developed and the developing world. Prevention of the spread of antibiotic resistance “from farm to fork” is one essential constituent in the One Health approach [189].

## 11 A look into the future

It is undisputed that collaborative efforts such as the “World Alliance Against Antibiotic Resistance”, and the “Joint Programming Initiative on Antimicrobial Resistance”, among others, are needed to tackle the hazards posed by antibiotic resistance [190]. 

For example, novel approaches in pharmaceutical research such as overcoming the intrinsic resistance of GNB by designing glycopeptide analogues which can permeate the outer membrane of GNB can lead to interesting therapeutic options, which may help counteract the impact antibiotic resistance has on the therapy options of immunosuppressed patients [191].

In addition, there is need for alternative approaches focussing on infection prevention, which make the use of antimicrobial agents unnecessary. Novel designs for equipment and fittings which serve as a reservoir for GNB, are of special interest. These include plumbing systems, sinks and sink drains, water outlets, medical devices as well as washing machines (cf. [55]). 

*“At the present time, our best defense against (MDRO) … remains old-fashioned, stringent infection control measures combined with the application of effective antimicrobial stewardship”* [192]. Although this sentence sounds like an easy “take home message” it does implicate that infection control measures are only as good as they are implemented stringently and concomitantly with antimicrobial stewardship, which is difficult but not impossible to achieve. Thus, for example, endemic MDR *A. baumannii* in ICUs of Chungnam National University Hospital in Daejeon, South Korea, was brought under control by introducing an enforcing antimicrobial stewardship along with comprehensive, intensified infection control measures, including cohorting of patients in designated areas and promotion and monitoring of hand hygiene and environmental cleaning and disinfection protocols [193].

It has also been shown that *Organizational Culture* (staff engagement) is positively correlated with prevention attitudes and compliance with contact precaution protocols and negatively correlated with carbapenem-resistant *Enterobacteriaceae* acquisition rates [194]. The principle of *“Patient Safety First”* should be laid down in quality assurance regulations and include preventing antibiotic resistance and handling of MDRO, respectively, but it must also be part of a *culture* of patient safety. It cannot be overemphasized that awareness, rules and regulations will not automatically result in a change in routines nor in skilled and effective performance of the necessary chores.

Again, theses measures do not specifically target GNB. However, as we search for strategies, it becomes apparent that detailed investigations on Gram-negative bacteria, their intrinsic resistance, the spread of acquired resistance, the difficulties in reliable and rapid diagnosis, their ubiquitous reservoirs have forced all those involved with the prevention of infection to develop a “global perspective” and a “One Health perspective” which will eventually benefit all efforts to curtail antibiotic resistance.

Meanwhile – shortly before publication of this paper – WHO has published a *Global priority list of antibiotic-resistant bacteria to guide research, discovery, and development of new antibiotics* [195]. It addresses the urgent need for research efforts for GNB-MDR as they pose a particular threat to hospitals, nursing homes and patients requiring ventilation and catheterization. They are to be considered the most critical group of multidrug-resistant bacteria.

## 12 Conclusions

In view of these developments, the authors have agreed on the following principle key points to be addressed to various target groups where applicable:

Policy makers

- Issue guidelines
- Support efforts in research and development (see below)
- Establish legal framework for mandatory implementation of guidelines
- Take care of water and sanitation and waste management
- Support education and training for the general public
- Support adequate education and training for medical students and personnel

General public

- Increase knowledge about infection prevention and prudent use of antibiotics

Resident physicians/dentists

- Comply with antibiotic stewardship
- Substitute antibiotics by analogous or even higher effective measures, i.e. antiseptics, probiotics, and bacteriophages

Hospital and medical facilities

- Create a patient safety culture
- Implement prevention and infection control precautions
- Implement novel strategies for building sink/sink drainage and sewage systems
- Implement adequate design of patient rooms and wards

Healthcare professionals and caregivers

- Comply with rules and regulations
- Promote patient empowerment
- Take advantage of training and education

Infection control preventionists

- Devise guidelines and recommendations
- Identify research gaps
- Monitor implementation of guidelines

Industry

- Research & development (pharmaceuticals, medical devices, architectural, disinfectants, fittings and furnishings)

Agriculture

- Reduce antibiotic use in farming/animal breeding
- Implement sanitation of stable environment
- Eliminate and reduce pathogenic bacteria by cleaning and disinfection
- Enhance individual defense mechanisms and monitoring of resistance status

Research & development

- Develop new antibiotics against MDRO-GNB
- Develop safer and more effective biocides against MDRO-GNB
- Provide means for rapid diagnosis of sepsis and MDRO-GNB-associated sepsis
- Explore role of specific dietary and nutritional interventions in reducing/ suppressing gut colonization by MDR-GNBs
  - Role of bael fruit/yoghurt (curd)
  - Micronutrients: Vitamins/minerals
- Explore role of interventions to reduce or eliminate gut colonization with MDR-GNBs:
  - Role of probiotics
  - Rifaximin
  - Oral colistin
  - Oral neomycin
  - Bowel wash
- Explore feasibility of vaccines against certain MDR-GNB (e.g. against Klebsiella)
- Explore role of pre-operative or pre-intervention (e.g. transplantation) screening for MDR-GNB in reducing post-intervention infective morbidity and mortality
- Develop new strategies in the design and infrastructure of water sanitation and hygiene programs for various settings.

## Notes

### Origin

This paper is based on the proceedings of the Rudolf Schülke Symposium ”Worldwide Significance of Gram-Negative Antibiotic Resistant Rods: Epidemiology, Prevention and Control Strategies“ held in Hamburg, 26 and 27 November 2015. All presentations are available for download from http://www.rudolf-schuelke-stiftung.de/rudolf-schuelke-stiftung/aktuelles/news.php. 

Symposium participants: S. Bhattacharya, B. Christiansen, M. Exner, J. Gebel, P. Goroncy-Bermes, P. Hartemann, P. Heeg, C. Ilschner, A. Kramer, E. Larson, W. Merkens, M. Mielke, P. Oltmanns, B. Ross, M. Rotter, R. Schmithausen, H. G. Sonntag, M. Trautmann.

### Competing interests

The authors declare that they have no competing interests. 

## Figures and Tables

**Table 1:**
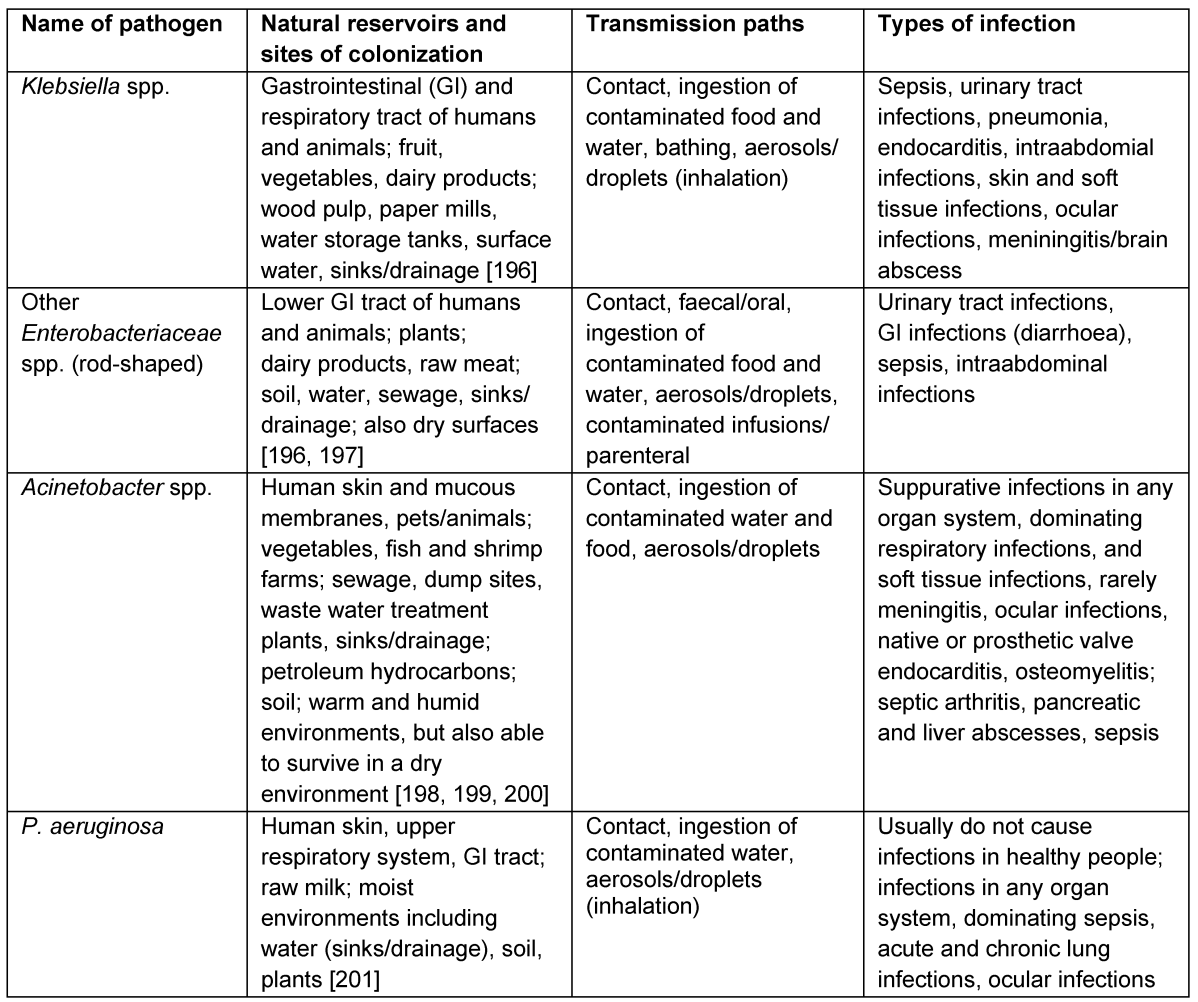
Examples for frequent natural reservoirs and sites of colonization, transmission paths and types of infection of selected Gram-negative bacteria
